# A MACHINE LEARNING MODEL FOR THE PROGNOSIS OF ALZHEIMER’S DISEASE DEMENTIA AT THE MILD COGNITIVE IMPAIRMENT STAGE USING MITOCHONDRIAL METHYLCYTOSINES AS NOVEL BLOOD‐BASED BIOMARKERS

**DOI:** 10.1002/alz.091321

**Published:** 2025-01-09

**Authors:** Jose Luis Mosquera, Marta Blanch, Beatriz Fontal, Pau Martí, Paula Ferrer, Carla Trapero, Nuria Rojo, Inma Rico, Jaume Campdelacreu, Joan Bello López, Alberto Lleo, Juan Fortea, Pablo Martinez‐Lage, Adrià Tort‐Merino, Raquel Sanchez‐Valle, Carlos Cruchaga, Christopher J Fowler, Simon M. Laws, Courosh Mehanian, Ramon Rene‐Ramirez, Jordi Gascon‐Bayarri, Marta Barrachina

**Affiliations:** ^1^ ADmit Therapeutics SL, Barcelona, Barcelona Spain; ^2^ Functional Unit of Dementia, Service of Neurology, Bellvitge University Hospital, Bellvitge Biomedical Research Institute, IDIBELL, Barcelona Spain; ^3^ Neurology Service, Complex Hospitalari Moisès Broggi. L’Hospitalet/Sant Joan Despí, Barcelona Spain; ^4^ Center for Biomedical Investigation Network for Neurodegenerative Diseases (CIBERNED), Madrid Spain; ^5^ Department of Neurology, Institut d'Investigacions Biomèdiques Sant Pau ‐ Hospital de Sant Pau, Universitat Autònoma de Barcelona, Hospital de la Santa Creu i Sant Pau, Barcelona Spain; ^6^ Centre of Biomedical Investigation Network for Neurodegenerative Diseases (CIBERNED), Madrid Spain; ^7^ Center for Research and Memory Clinic. CITA‐alzheimer Foundation, San Sebastian Spain; ^8^ Alzheimer’s disease and other cognitive disorders Unit. Hospital Clínic de Barcelona. Fundació de Recerca Clínic Barcelona – IDIBAPS. University of Barcelona, Barcelona Spain; ^9^ Hospital Clínic de Barcelona ‐ Fundació de Recerca Clínic Barcelona – IDIBAPS ‐ University of Barcelona, Barcelona, Catalonia Spain; ^10^ Washington University in St. Louis, School of Medicine, St. Louis, MO USA; ^11^ The Florey Institute of Neuroscience and Mental Health, The University of Melbourne, Parkville, VIC Australia; ^12^ Collaborative Genomics and Translation Group, School of Medical and Health Sciences, Edith Cowan University, Joondalup, Western Australia Australia; ^13^ Global Health Labs, Bellevue, WA USA; ^14^ University of Oregon, Eugene, OR USA

## Abstract

**Background:**

Prediction of progression to Alzheimer’s Disease Dementia (ADD) at the Mild Cognitive Impairment (MCI) stage is an unmet medical need.

Mitochondrial dysfunction in Alzheimer’s Disease at the brain and systemic level has been extensively described. Our previous studies showed an altered mtDNA methylation pattern throughout AD progression in human postmortem brains (Blanch et al. 2016). Similar results were found in blood samples from MCI patients (Stoccoro et al. 2022).

ADmit has developed an epigenetic Next‐Generation Sequencing approach for identifying differential mtDNA methylation patterns in blood samples from MCI patients. The methylation measures, along with non‐invasive clinical features, have been integrated into a classification model that predicts the probability of progression to ADD.

**Method:**

A total of 600 subjects were recruited from longitudinal prospective clinical studies: AIBL Consortium (n=248), Hospital Universitari Bellvitge (n=125), CITA‐Alzheimer Foundation (n=75), Hospital Clínic Barcelona (n=79), Knight‐ADRC (n=64), Hospital Sant Pau (n=5), and Hospital General Hospitalet (n=4). The analysis was focused on blood samples from the baseline visit for Cognitively Normal (CN, CDR=0, n=168) and MCI subjects (CDR=0.5, n=432) with a clinical follow‐up between 3 and 16 years. The model classifies the subject as CN, MCI that does not progress to ADD, and MCI that does progress to ADD, using over 200 methylation measures and some clinical variables without including the AbPET scan. An MCI patient was considered progressed to ADD when their CDR shifted to ≥ 1, or there was a ≥ 2‐point deterioration in CDR‐SOB, or ≥ 1‐point deterioration in at least four instrumental daily life activities. The dataset was split in two subsets for training and for testing (80:20). Several methods were explored in previous analyses. Random Forest showed a superior performance through a triply replicated 10‐fold Cross‐Validation and feature selection process.

**Result:**

The predictions on the testing data are: Accuracy=0.832 (CI95% 0.752‐0.894), Kappa=0.711, F1‐score=0.853, Sensitivity=0.951, Specificity=0.707, PPV=0.773, NPV=0.932, and an AUC=0.775.

**Conclusion:**

The present classification model performs accurate classification of ADD at MCI stage up to 14 years before the appearance of clinical signs of dementia and represents a promising minimally‐invasive prognostic tool. This is the first blood‐genomic (mitochondria) prediction model.

